# *Staphylococcus aureus* can degrade exogenous fatty acids through β-oxidation

**DOI:** 10.1128/mbio.00609-26

**Published:** 2026-04-20

**Authors:** Cindy Menjivar, Clarissa Shoffler, Christopher Petucci, Jeffrey L. Bose

**Affiliations:** 1 Department of Microbiology, Molecular Genetics, and Immunology, University of Kansas Medical Center, Kansas City, Kansas, USA; 2 Penn Metabolomics Core, Cardiovascular Institute, Department of Medicine, Perelman School of Medicine at the University of Pennsylvania, Philadelphia, Pennsylvania, USA; NYU Langone Health, New York, New York, USA

**Keywords:** β-oxidation, MRSA, fatty acid, metabolism

## Abstract

*Staphylococcus aureus* has long been thought to lack a functional fatty acid degradation (Fad) pathway despite the presence of a locus annotated with the necessary *fad* genes; however, two recent studies have examined the *fad* operon for the first time. While they provided key insights about the system and both identified that exogenous fatty acids can be added to coenzyme A to produce an acyl-CoA, neither identified whether degradation occurs. The *fad* operon contains two sizeable intergenic regions even though the *fadXDEBA* genes can be expressed on a single polycistronic mRNA under the control of the *fadX* promoter. We hypothesized that these intergenic regions could be a point of regulation that prevents full Fad function; therefore, we generated a new *S. aureus* strain where the two intergenic regions of the *fadXDEBA* operon were deleted. Using mass spectrometry analysis with this strain, we detected degradation products from both palmitic acid and oleic acid, a saturated and unsaturated fatty acid, respectively. The products were absent in a *fadXDEBA* mutant. These data demonstrate that the *S. aureus* fatty acid degradation system is functional and can degrade exogenous fatty acids.

**IMPORTANCE:**

There have been limited studies on the fatty acid degradation (Fad) pathway of *Staphylococcus aureus*. The *fadXDEBA* operon has been shown to contain all the genes necessary for β-oxidation, and the FadD and FadBA proteins have been shown to perform their canonical functions. This study demonstrates that the full *S. aureus* Fad pathway is functional and capable of degrading exogenous fatty acids. These data bring to light a second exogenous fatty acid utilization pathway, expanding our knowledge on how this pathogen metabolizes environmental fatty acids.

## OBSERVATION

*Staphylococcus aureus* is a pathogen that can cause an array of diseases (1). While only colonizing ~30% of the population asymptomatically (2), its fine-tuned regulation of virulence factors allows *S. aureus* to circumvent host defenses and opportunistically cause infections. The plethora of virulence factors work in tandem with the pathogen’s versatile metabolism to ensure environmental adaptability.

**Fig 1:**
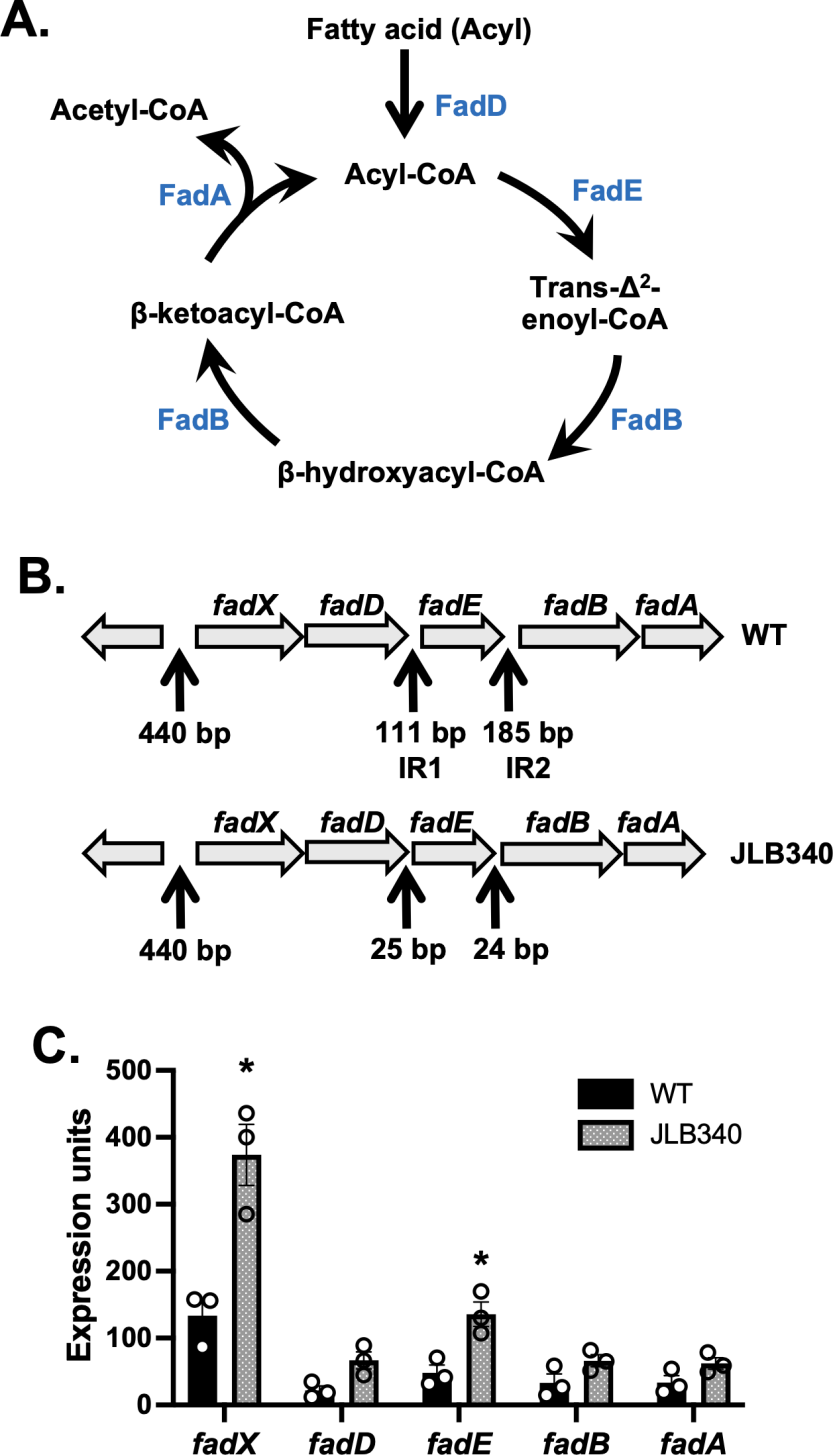
Deletion of *fad* IR1 and IR2. (**A**) Schematic of the Fad pathway. Acyl-CoA molecules decrease by two carbons every cycle with release of acetyl-CoA. (**B**) Schematic of the *fadXDEBA* operon in *S. aure*us. “440 bp” is the spacing between *fadX* and the upstream gene and contains the promoter. (Top) Intergenic regions (IR1 and IR2) include size (bp) in wild type. (Bottom) Deletion of IR1 and IR2 and spacing remaining between genes (bp) in JLB340. (**C**) qRT-PCR of *fad* genes in wild type and JLB340 grown to 7 hours in TSB without glucose and supplemented with 500 μM palmitic acid. Each symbol is a sample, and the column is the mean (*n* = 3) with SEM, while “*” indicates a significant difference (*P* < 0.05) by Welch’s *t*-test.

Pathogens encounter exogenous fatty acids (exoFAs) in a variety of host sites that could supplement endogenous production (3–5). For example, gram-positive bacteria utilize a Fak system to incorporate exoFAs into lipids. This supplements the endogenous fatty acid synthesis (FASII) pathway, aiding in membrane homeostasis (6, 7) and contributing to resistance against FASII inhibitors (8, 9). ExoFAs can also be a carbon source through fatty acid degradation (Fad), commonly called β-oxidation. The canonical Fad pathway metabolizes exoFAs in a cyclic manner, resulting in the release of the two-carbon metabolite, acetyl-CoA, per cycle (Fig. 1A) (10). *S. aureus* has long been thought to lack this ability based largely on a single report (11); however, two recent studies challenge this dogma (12, 13). Both studies provided evidence that *S. aureus* possesses a Fad pathway that is under strong regulatory control, but neither was able to identify a functional system beyond the first step, conversion of an exoFA to its acyl-CoA derivative. The fundamental question of whether *S. aureus* is capable of degrading fatty acids has remained. In this study, we demonstrate that *S. aureus* has the capacity to degrade fatty acids through the Fad pathway.

Previously, while characterizing the *S. aureus* Fad system, we noted two intergenic regions (IR1 and IR2) within the *fadXDEBA* operon (Fig. 1B). Despite the possibility of these regions containing alternative promoters, we were unable to detect promoter activity from the IRs under our conditions, and we demonstrated the *fadXDEBA* operon can be expressed on a single polycistronic mRNA (13). This was consistent with an RNA analysis performed by another group that demonstrated the *fad* genes responded similarly in 44 tested conditions (14). Thus, the importance of these IRs remains uncertain. While we and others have demonstrated that the first step for β-oxidation occurs (12, 13), our previous attempts to identify Fad products in our wild-type *S. aureus* strain were unsuccessful (13) (Fig. S1). As a result, we hypothesized the two intergenic regions could be areas of undescribed Fad regulation.

To investigate the impact of the IRs in Fad functionality, we generated an *S. aureus* mutant (JLB340) where the two IRs of the *fad* operon were deleted while leaving the native Shine-Dalgarno sites intact (Fig. 1B; Fig. S2). We first ensured that *fadXDEBA* was still expressed in TSB without glucose and supplemented with 500 μM palmitic acid, our previous growth condition to detect Fad products (Fig. 1C). We observed *fad* expression in both strains with slightly higher mRNA abundance in the JLB340 strain compared to WT. Moreover, the relative expression pattern for each gene mirrored that of WT.

**Fig 2:**
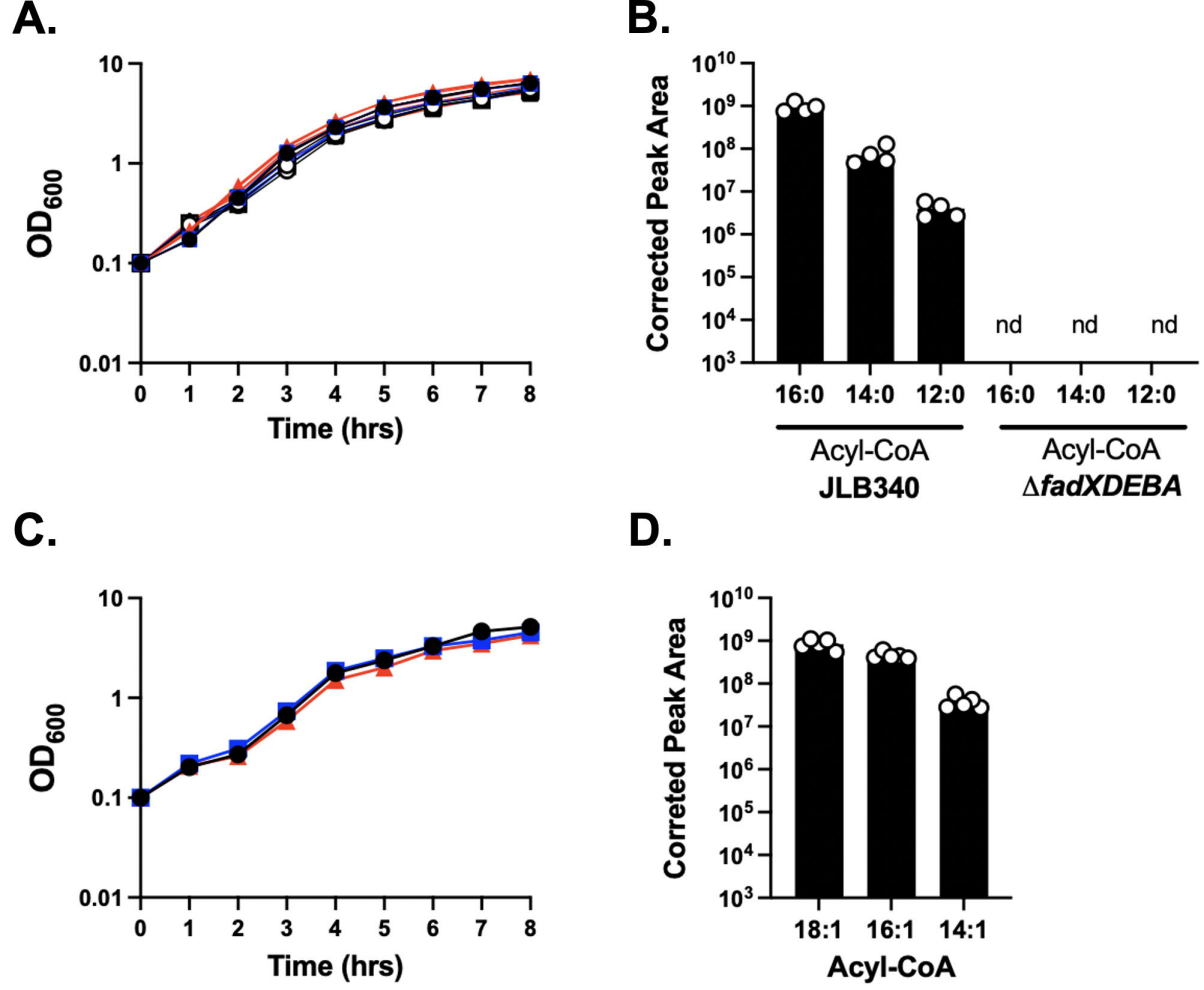
*S. aureus* can degrade exogenous palmitic and oleic acid. (**A**) Wild type (WT, black), JLB340 (blue)*,* and Δ*fadXDEBA* mutant (red) were grown 8 hours at a 1:10 media-to-flask ratio in TSB without (solid symbols) or supplemented with 500 μM palmitic acid (open symbols). Symbols represent the mean (*n* = 3) with SEM. Error bars are present and may be smaller than symbols. (**B**) The JLB340 strain and the Δ*fadXDEBA* mutant were grown in TSB without glucose for 7.5 hours total, with 500 μM [^13^C]palmitic acid (16:0) added after 4 hours of growth. Acyl-CoAs in cells were determined by mass spectrometry and corrected peak area determined for [^13^C]acyl-CoA species of each carbon length. (**C**) WT, JLB340*,* and Δ*fadXDEBA* mutant were grown as in panel A except with 314 μM oleic acid. Symbols represent the mean (*n* = 3) with SEM. Error bars are present and may be smaller than symbols. (**D**) JLB340 was grown in TSB without glucose for 7.5 hours total, with 314 μM [^13^C]oleic acid (18:1) added after 4 hours of growth. Acyl-CoAs with unsaturated fatty acids in cells were determined by mass spectrometry and corrected peak area for detected [^13^C]acyl-CoA of each carbon length. For panels **B** and **D**, each symbol is a sample, and the column is the mean (*n* = 5) with SEM. nd, not detected.

The first step of the Fad pathway is the conversion of an exoFA to an acyl-CoA by FadD, which is then processed by the Fad cycle where it releases acetyl-CoA (Fig. 1A). Thus, a truncated acyl-CoA or released acetyl-CoA serves as a marker for Fad activity. To test whether deletion of IR1 and IR2 would remove the block preventing degradation, we grew JLB340 and the Δ*fadXDEBA* mutant to mid-exponential phase and spiked the culture with [^13^C]palmitic acid with every carbon labeled. Both strains grow similarly to wild type in the absence or presence of palmitic acid (Fig. 2A). Deletion of IR1 and IR2 in JLB340 allowed the full Fad cycle to proceed as we were able to detect [^13^C]palmitoyl-CoA (16:0) molecules along with [^13^C]myristoyl-CoA (14:0) and [^13^C]lauroyl-CoA (12:0) (Fig. 2B; Fig. S3). Subsequent degradation products were also present based on the determined retention times, though they did not reach above our signal-to-noise ratio cutoff (Fig. S3). In addition, the molecule peaks were not present in control samples, i.e., samples with no labeled palmitic acid (not shown). [^13^C]acetyl-CoA was detected in two of our samples and was, on average, <3% of the total acetyl-CoA. These products were specific to Fad since these labeled metabolites were absent in the Δ*fadXDEBA* mutant (Fig. 2B). We also detected low-abundance unlabeled acyl-CoAs that likely come from endogenous sources or the media whose production was also Fad dependent as they were absent in the Δ*fadXDEBA* mutant (not shown). Since *S. aureus* commonly encounters the unsaturated fatty acid oleic acid (C18:1) in the host, we tested the ability of *S. aureus* to degrade it as well. We first confirmed that JLB340 grew similar to wild type in the presence of oleic acid (Fig. 2C). Analysis of the cells for [^13^C]acyl-CoA molecules containing unsaturated fatty acids revealed the conversion of oleic acid to an acyl-CoA ([^13^C]oleoyl-CoA) as well as two rounds of degradation (Fig. 2D). In this case, we also detected labeled acetyl-CoA in three of five samples that were on average 0.5% of the total acetyl-CoA levels. These data demonstrate the *S. aureus* Fad pathway is functional and can degrade exogenous fatty acids.

*S. aureus* has long been thought to be incapable of performing β-oxidation, a common metabolic pathway. This notion was widely accepted due to a previous unsuccessful attempt at detecting degradation activity (7) and the fact that Biocyc and KEGG pathway analysis annotated the *S. aureus* Fad pathway as incomplete. This was surprising due to this pathogen’s known versatile metabolism. In this current study, we demonstrate that *S. aureus* is capable of degrading fatty acids. However, this requires removal of IR1 and IR2, further supporting our hypothesis that these regions within the *fadXDEBA* operon possess regulatory roles that further dictate the function of the Fad system. The question remains as to how the IRs impact Fad function. Our hypothesis is that one or both IRs are points of post-transcriptional regulation. There are no obvious stem loops in either IR that could serve as transcription terminators, and previous studies (13, 14) support the notion that all the *fad* genes can be expressed on a single transcript. A study suggested the presence of a non-coding RNA produced from IR2 that is antisense to *fadEDX* (15); however, an analysis of RNAseq from 44 conditions did not reveal the presence of this RNA (14) and our attempts using RT-PCR to directly detect it were unsuccessful. Thus, this is unlikely the cause of Fad inhibition when IR1 and IR2 are present. Another possibility is that an unknown RNA-RNA interaction occurs at one or both sites. There is precedence for this as Fad in *Vibrio cholerae* was recently found to be controlled by such a mechanism (16). Further studies are needed to understand the multilayered regulation of the *S. aureus* Fad pathway. Now that two exogenous fatty acid utilization systems are shown to be functional in *S. aureus*, future work will be needed to determine if these two systems intersect and how they contribute to overall metabolism. Our findings, along with others, are redefining fatty acid metabolism in *S. aureus* by showing this bacterium is capable of degrading exogenous fatty acids.

## SUPPLEMENTAL MATERIAL

Supplemental material mbio.00609-26-s0001.pdf Supplemental figures, tables, and methods,

ASM does not own the copyrights to Supplemental Material that may be linked to, or accessed through, an article. The authors have granted ASM a non-exclusive, world-wide license to publish the Supplemental Material files. Please contact the corresponding author directly for reuse.
