# Supplementary material for: Staphylococcus aureus can degrade exogenous fatty acids through β-oxidation
Source: mBio. 2026 Apr 20;17(5):e00609-26. doi: 10.1128/mbio.00609-26 (PMC13170264; doi:10.1128/mbio.00609-26)
Supplement: Supplemental material — Supplemental figures, tables, and methods, [file mbio.00609-26-s0001.pdf]

Supplementary Information:

Staphylococcus aureus can degrade exogenous fatty acids through β-oxidation

Cindy Menjivar[1], Clarissa Shoffler[2], Christopher Petucci[2], and Jeffrey L. Bose[1*]

[1]Department of Microbiology, Molecular Genetics, and Immunology, University of Kansas Medical Center, Kansas City, Kansas, USA

[2]Penn Metabolomics Core, Cardiovascular Institute, Department of Medicine, Perelman School of Medicine at the University of Pennsylvania, Philadelphia, Pennsylvania, USA

*Corresponding Author

E-mail: jbose@kumc.edu

Figure S1: WT *S. aureus* cannot degrade fatty acids

Figure S2: Sequence of IR1 and IR2

Figure S3: MS spectra of degradation products and standards

Materials and Methods

Table S1: Bacterial strains and plasmids

Table S2: Oligonucleotides

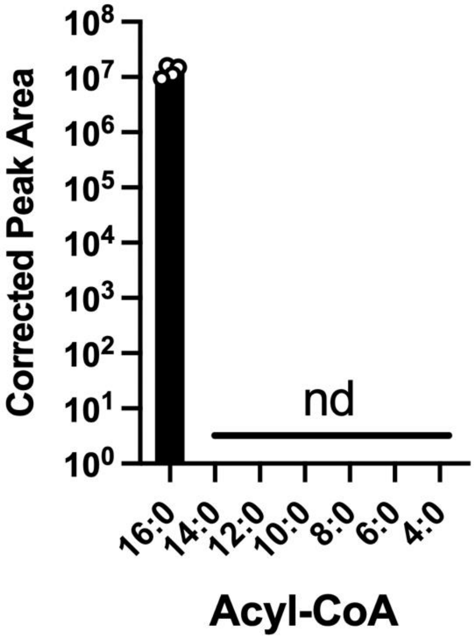

Supplemental Figure 1. Wild-type strain grown in TSB without glucose for 4 hours total, with 500 μM [^{13}C]palmitic acid added after 2 hours of growth. Cell pellets were analyzed by mass spectrometry. Acyl-CoAs in cells were determined by mass spectrometry and corrected peak area determined for [^{13}C]acyl-CoA species of each carbon length. Each symbol is a sample, and the column is the mean (n=4) with SEM. nd=not detected. Mass spectrometry data was reanalyzed from Menjivar 2025.

A.

IR1

TAAAGGTGATAAAAATTTTTGACATTTAGTGTAAGCGTTT
ACAAATAAAGCGTGTTGTTTTTGAATTAAATGCATTTCAC
ATTAGTATTCATATTATTTTTAGGAGGAATTTATATG

B.

IR2

TAAATAGCAAATAATTATATGAGATGCATTAATTTCACTA
AAAAAGACTTATTTTAAGCATAAAGCTTTTTCCTTAAATA
AGAGGCTAAGATGACTGTCAAAGATACTTAATTAATTTTA
TAAAATAGCAACGTTATTCCAATTATCTTAATGGTTATCT
TATCCTCAACTAAATTGGAGGAATCACTATG

Supplemental Figure 2. Deleted sequences of IR1 and IR2. Schematic showing the complete sequences of **A)** IR1 and **B)** IR2. Nucleotides between the underlined sequences were deleted. The red text denotes stop codon of upstream open reading frame and green highlight is the start of next open reading frame.

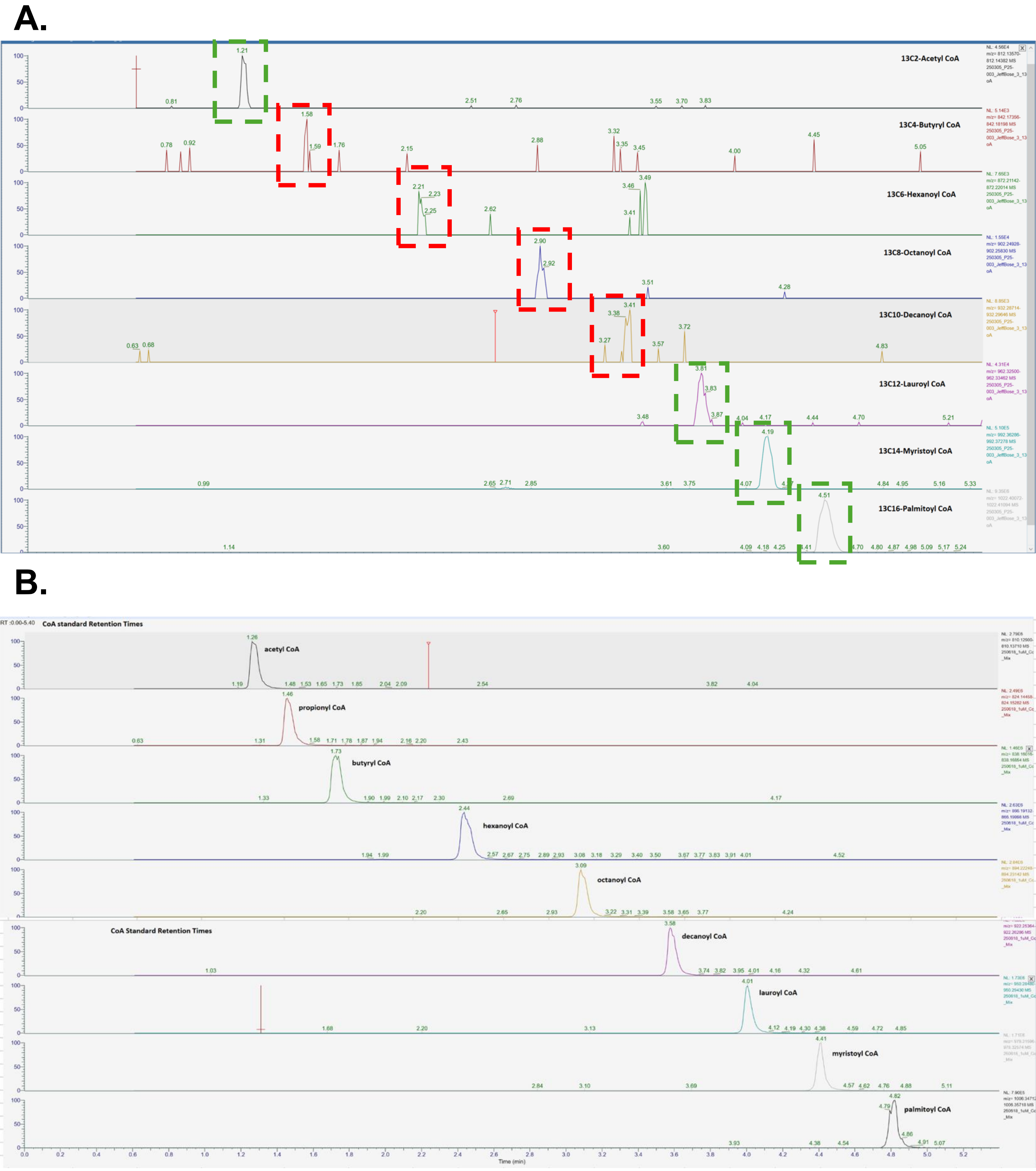

Supplemental Figure 3. Degradation products from palmitic acid. A) JLB340 was grown in TSB without glucose for 7 hours total, with 500 µM [^{13}C]palmitic (16:0) added after 5 hours of growth. Cell pellets were analyzed by mass spectrometry. Boxes denote those products that were greater than the signal to noise ratio threshold (green) and those that were not (red). Sample shown is representative of multiple samples and is a separate experiment than Figure 2. **B)** Unlabeled standards purchased from Sigma Aldrich or Avanti Polar Lipids.

Materials and Methods

Bacterial strains and growth conditions.

For all experiments, *S. aureus* strains (Table S1) were cultured from -80°C freezer stocks on tryptic soy agar (TSA) and sub-cultured overnight in 3 mL of tryptic soy broth (TSB). TSB was supplemented with chloramphenicol (10 µg ml^{-1}) when necessary. Unless otherwise stated, overnight cultures were diluted to OD_{600} = 0.1 in 12.5 mL of filter-sterilized TSB (with no dextrose, BD cat#: 286210) in 125-ml flasks. *Escherichia coli* DH5α was used for cloning and was grown in 4 mL lysogeny broth (LB) supplemented with ampicillin (100 µg ml^{-1}). Palmitic acid was resuspended in chloroform to make a stock solution of 250 mM.

Cloning.

PCR was performed using oligos synthesized by Integrated DNA Technologies using KOD DNA Polymerase kit (Sigma Aldrich). Primers are listed in Table S2. Plasmids were isolated using the Promega Wizard *Plus* SV Minipreps DNA Purification System (Promega, Madison, WI). DNA digests and ligations were performed using enzymes from New England Biolabs and were cleaned with Zymo Research Clean and Concentrator kits between steps. Sequencing of inserts was performed by ACGT, Inc.

Plasmids and deletion strains JLB334 and JLB340 (Table S1) used genomic DNA from AH1263 as the template. To generate pCM6 for the deletion of IR1, primer pair CM11 and CM12 were used to amplify the upstream of gene *fadE* and cloned into the EcoRI and KpnI sites of pJB38. Simultaneously, primer pair CM13 and CM14 were used to amplify the downstream of gene *fadE* and cloned into the KpnI and SalI sites of pJB38.

To generate pCM7 for the deletion of IR2, primer pair CM15 and CM16 were used to amplify the upstream of gene *fadB* and cloned into EcoRI and XbaI sites of pJB38. Simultaneously, primer pair CM17 and CM18 were to amplify the downstream of gene *fadB* and cloned into XbaI and SalI sites of pJB38.

All plasmid constructions were performed in *E. coli* and then transformed into *S. aureus* RN4220. Plasmids were transduced into AH1263 using φ11. Allelic exchange was performed first with pCM7 to generate JLB334, which was confirmed after DNA isolation by PCR using primer pair CM15 and CM18. JLB334 was then transduced with pCM6, followed by allelic exchange. Deletion of IR1 was confirmed after DNA isolation with primer pair CM11 and CM14 via PCR and IR2 was confirmed again with CM15 and CM18. Whole genome sequencing of JLB340 was the performed by SeqCenter, LLC.

Real-time-PCR.

Cultures were grown for 7 hours (exponential phase) at which 1 mL of culture was harvested at 4,500 x *g* for 5 min at 4 °C. The supernatant was removed, and the pellet frozen at -80°C. After thawing on ice, the pellet was resuspended in TE buffer and transferred to a Lysing Matrix B Tube (MP Biomedicals), and cells were subjected to lysis using the FastPrep-24 5G homogenizer (MP Biomedicals). Total RNA was extracted using the RNeasy Mini Kit (Qiagen) and treated with DNase (TURBO DNA-*free* Kit (Invitrogen)). RNA was quantified using a NanoDrop One (Thermo Fisher Scientific) and cDNA was synthesized using the QuantiTect Reverse Transcription Kit (Qiagen) with 500 ng of total RNA used as the template. The cDNA was diluted 25-fold in nuclease-free H_2O. A reaction mixture containing FastStart Essential DNA Green Master (Roche),

primers (5 μM each, designated as RT-*fad*F for forward and RT-*fad*R for reverse), and H_2O was added to the diluted cDNA. Finally, 19 μL of reaction mixture combined with cDNA was aliquoted in triplicate into a 96-well plate and amplification was performed using a LightCycler96 (Roche). The calibrator for calculations was *sigA* (*rpoD*).

Growth Curves.

Overnight cultures were diluted to OD_{600} = 0.1 in 25 mL of filter-sterilized TSB (with no dextrose, BD cat#: 286210) in 250-mL flasks. TSB was supplemented with 500 μM palmitic acid (Thermo Scientific cat#: 129702500) and 314 μM oleic acid (Fisher Scientific cat#: AA3199706) when appropriate. OD_{600} readings were taken every hour for 8 hours using a Thermo Fisher Scientific GENESYS 10S UV/VIS spectrophotometer.

Mass Spectrometry Growth.

Overnight cultures were diluted to OD_{600} = 0.1 in 15.5 mL of filter-sterilized TSB (with no dextrose, BD cat#: 286210) in 125-mL flasks and grown for 4 hours. At 4 hours, cultures were spiked with 500 μM [^{13}C]palmitic acid (Cambridge Isotopes Laboratories, Inc. cat#: CLM-409-0.5) or 314 μM [^{13}C]oleic acid (Cambridge Isotopes Laboratories, Inc. cat#: CLM-460-0.1) and grown for another 3.5 hours. After 7.5 hours of growth, 12.5 mL of culture was harvested at 4,500 x *g* for 10 min at 4 °C. The supernatant was removed, and the pellets were immediately frozen at -80 °C.

Mass Spectrometry.

Frozen pellets of *S. aureus* were homogenized in 100 μL of 80% methanol in 0.5

mL Precellys tough microorganism lysing tubes with glass beads at 6800 rpm (3 x 30s, 20s pause) at 4 °C on a Precellys homogenizer (Bertin Technologies, Rockville MD). The homogenates were transferred to 1.5 mL Eppendorf tubes with 400 µL of methanol, vortexed, and centrifuged at 18,000 x g for 5 minutes at 4 °C. The supernatants were dried at 30 °C under nitrogen in a 96 well plate and reconstituted in 100 µL of 10 mM ammonium bicarbonate buffer at pH 9.5 for LC/MS. A 10 µL aliquot was injected on a Thermo Scientific Vanquish UHPLC to separate Coenzyme As (C2, C4, C6, C8, C10:1, C10, C12:1, C12, C14:1 C14, C16:1, C16, C18:1, C18) using a gradient from 100% A (10 mM ammonium bicarbonate pH 9.5)/0% B (ACN) to 20% B over 1.25 min to 95% B at 6.75 min followed by re-equilibration up to 13.8 min at 0.4 mL/min at 30 °C using a Waters Acquity BEH C18 2.1 x 50 mm, 1.7 µm column. A Thermo Scientific Orbitrap ID-X connected to the UHPLC was operated in positive electrospray ionization mode (3500 V, sheath gas flow 50 L/min, auxillary gas flow 10 L/min, sweep gas flow 1 L/min, vaporizer 350 °C, ion transfer tube 325 °C) and scanned from m/z 800-1100 at 120K resolution). 13C isotopologues were extracted from the total ion chromatograms, corrected for natural abundance using AccuCor, and corrected peak areas were used to determine %^{13}C incorporation into CoAs (peak area of labelled isotopologue/total area of all isotopologues x 100%).

Table S1. Bacterial strains and plasmids.

Strain or plasmid	Description[a]	Reference(s) or source
**Strains**		
Staphylococcus aureus		
AH1263	USA300 CA-MRSA strain LAC without LAC-p03, wild-type strain used for these studies	[1]
JLB333	AH1263 Δ*fadXDEBA*	[2]
JLB334	AH1263 Δ*fad* IR2 (*fadE-fadB*)	This study
JLB340	AH1263 Δ*fad* IR1/2 (*fadD-fadE* &*fadE-fadB*)	This study
RN4220	Highly transformable *S. aureus*	[3]
Escherichia coli		
DH5α	F^- Φ80*lacZ*ΔM15, Δ(*lacZYA-argF, U169, deoR, supE44, hsdR17, recA1, endA1, gyrA96, thi-1, relA1*)	[4]
**Plasmid**		
pCM6	Intergenic region between *fadD* and *fadE* genes (0228-0227) deletion plasmid	This study
pCM7	Intergenic region between *fadE* and *fadB* genes (0227-0226) deletion plasmid	This study
pJB38	Temperature-sensitive allelic exchange vector, Amp/Cm^R	[5]

a Amp^R and Cm^R denote ampicillin and chloramphenicol resistance cassettes.

Table S2. Oligonucleotides.

Oligonucleotides*	Sequence (5'-3')#	Reference or source
CM11	gtcgaattcCGCTACGTGCTAGTAATTTTAATCCTG	[2]
CM12	gctGGTACCTTTTTATCACCTTTAAAGTGTTTGCGAGAC	This study
CM13	gacggtaccAGGAGGAATTTATATG ACATTTGAAAAAG	This study
CM14	gaggtcgacGATCCGCTTCATCTACTTTGACATTAG	This study
CM15	gtcgaattcAGGCGATACTTGGGTTATCAATGG	[2]
CM16	cggtctagaTGCTATTTATACGAAAGCAGAATCTCC	This study
CM17	gagtctagaTAAATTGGAGGAATCACTATGACAATTAATAAAG	This study

CM18	gaggtcgacTACAGACACTGCAATATCTAAACCGAC	This study
*CM49 (RT-*fadB*F)	AAGGTGATGCCAAACTTTCC	[2]
*CM50 (RT-*fadB*R)	ATCGCATCAACTAACGCATC	[2]
*RT-*fadX*F	GCCTTGTCTGGAGAAGAACG	[2]
*RT-*fadX*R	GTTCACGACTCCCAACGAAT	[2]
*CM72 (RT-*fadD*F)	GTTCGTTGATGCACGTATCC	[2]
*CM73 (RT-*fadD*R)	TGAAATGCTTTGGCAGTCTC	[2]
*RT-*fadE*F	CCAAGGCGATACTTGGGTTA	[2]
*RT-*fadE*R	TAGGGCGTTAGGAACAATGC	[2]
*RT-*fadA*F	TGCATATGCGATTCCTGAAG	[2]
*RT-*fadA*R	TAACATTGCGCCTGTAGCAC	[2]
*JBSIGAF	AACTGAATCCAAGTGATCTTAGTG	[6]
*JBSIGAR	TCATCACCTTGTTCAATACGTTTG	[6]

* Denotes primers used for real-time PCR.

# Lowercase letters are those added for cloning purposes.

References

1. Boles, B.R., et al., *Identification of genes involved in polysaccharide-independent Staphylococcus aureus biofilm formation.* PLoS One, 2010. **5**(4): p. e10146.
2. Menjivar, C., et al., *Characterizing the Staphylococcus aureus fatty acid degradation operon.* J Bacteriol, 2025. **207**(8): p. e0008925.
3. Kreiswirth, B.N., et al., *The toxic shock syndrome exotoxin structural gene is not detectably transmitted by a prophage.* Nature, 1983. **305**(5936): p. 709-12.
4. Hanahan, D., *Studies on transformation of Escherichia coli with plasmids.* J Mol Biol, 1983. **166**(4): p. 557-80.
5. Bose, J.L., P.D. Fey, and K.W. Bayles, *Genetic Tools To Enhance the Study of Gene Function and Regulation in Staphylococcus aureus.* Appl Environ Microbiol, 2013. **79**(7): p. 2218-2224.
6. Lehman, M.K., et al., *Identification of the amino acids essential for LytSR-mediated signal transduction in Staphylococcus aureus and their roles in biofilm-specific gene expression.* Mol Microbiol, 2015. **95**(4): p. 723-37.
